# Editorial: Novel strategies for the prevention and treatment of foam cell formation and atherosclerosis

**DOI:** 10.3389/fcvm.2023.1333285

**Published:** 2023-11-22

**Authors:** Suowen Xu, Shaoyi Zheng, Xiu Liu, Coen van Solingen, Sijia Liang

**Affiliations:** ^1^ Department of Endocrinology, Institute of Endocrine and Metabolic Disease, The First Affiliated Hospital of USTC, Division of Life Sciences and Medicine, Clinical Research Hospital of Chinese Academy of Sciences (Hefei), University of Science and Technology of China, Hefei, China; ^2^ Department of Cardiovascular Surgery, Nanfang Hospital, Southern Medical University, Guangzhou, China; ^3^ NYU Cardiovascular Research Center, Leon H. Charney Division of Cardiology, Department of Medicine, New York University Langone Health, New York, NY, United States; ^4^ Department of Pharmacology, Cardiac and Cerebral Vascular Research Center, Sun Yat-sen University, Guangzhou, China

**Keywords:** atherosclerosis, foam cell formation, inflammation, lipid metabolism, novel strategies

**Editorial on the Research Topic** Novel strategies for the prevention and treatment of foam cell formation and atherosclerosis

## Introduction

Cardiovascular diseases (CVDs) remain the most prominent cause of death worldwide. Despite therapeutic advances, the rate of decline has slowed (1). It is therefore essential to investigate the underlying molecular mechanisms that improve our understanding of CVD to advance the health of all individuals and communities. This Editorial for the Research Topic *Novel Strategies for the Prevention and Treatment of Foam Cell Formation and Atherosclerosis* discusses the main findings of the published papers in this collection.

The most common cause of CVD is atherosclerosis, a condition in which imbalances in lipid metabolism and a maladaptive immune response drive the formation of plaques in the artery wall, which can ultimately lead to myocardial infarction and stroke. Foam cell formation is regarded as the hallmark of atherosclerosis. In this Research Topic, Shabani et al. investigated factors influencing cardiovascular mortality in heart failure patients, emphasizing the significance of pre-diagnostic long-term risk factor exposure. The findings highlight age, cardiac arrest, myocardial infarction, diabetes, QRS duration, HDL levels, cumulative cholesterol and glucose exposure, NT-proBNP, left ventricular mass, and statin use as crucial factors when predicting cardiovascular mortality. The study suggests that assessing cumulative cholesterol and glucose exposure, rather than relying solely on current measurements, will enhance predictive accuracy in heart failure mortality. Although statistical significance was not reached, the study paves the way for more personalized care and prevention measures in managing heart failure patients. Further studies are needed to validate and expand upon these valuable insights.

Atherosclerosis is a chronic progressive inflammatory form triggered by cholesterol-rich lipoproteins and other noxious factors (2). Activated neutrophils undergoing enhanced formation of neutrophil extracellular traps (NETs) have been reported to be associated with inflammatory response and atherosclerosis (3). Deletion of enzyme peptidylarginine deiminase 4 (PAD4), which has been highly linked to NET formation, is supposed to play a protective role against inflammation and atherosclerosis based on the absence of NETs (4). Surprisingly, Paunel-Görgülü et al. report that PAD4 deficiency in bone marrow cells limited lipid accumulation, plaque instability, and atherosclerotic lesion development, accompanied by increased pro-inflammatory M1-like macrophages (Paunel-Görgülü et al.). The authors assume that the reduced lipid accumulation in mice with PAD4 deficiency was associated with the reduced M2-like macrophage proportion, as they have previously shown that M2 macrophages incorporate more lipids than M1 macrophages. By contrast, similar studies by previous investigators demonstrated pro-inflammatory properties of PAD4 and NETs (4, 5). The reasons for the different results are currently unknown; however, these data suggest that the role of inflammation in atherogenesis may contain layers that remain to be unveiled. As such, although PAD4 may represent a therapeutic target for atherosclerosis amelioration based on the regulation of plaque progression, its precise role in atherosclerosis and the underlying mechanisms should be further explored.

The study by Kettunen et al. explores the link between the 9p21.3 chromosomal region and atherosclerosis, a major contributor to coronary artery disease (CAD). Using murine models, their study demonstrates that deleting the murine ortholog of the human 9p21.3 locus amplifies atherosclerosis and enhances macrophage proinflammatory activity. These findings shed light on the potential mechanisms underlying the genetic risk locus's impact on atherosclerosis. Notably, the study establishes a crucial link between genetics and inflammation-driven pathways in the development of atherosclerosis, providing valuable insights for comprehending and potentially treating CAD. Subsequent research endeavors hold the promise of unveiling innovative therapeutic strategies aimed at targeting this specific risk locus.

The Mini Review by Maxfield et al. summarizes recent studies that have unraveled novel mechanisms for the digestion of lipoproteins by macrophages in artery vessel walls. The authors discuss the control of the binding of oxidized lipoproteins by class A scavenger receptor (SRA) and CD26 through the induced expression of microRNA-204. As such, when the mechanism of action is unraveled in enough detail, microRNA-204 may represent a potential therapeutic avenue to control atherosclerotic development. Another topic considered in this Mini Review is three-dimensional electron microscopy studies describing the interactions between macrophages and aggregated lipoproteins. Electron microscopic images shown in this work show how macrophages directly bind to aggregated LDL, further detailing the formation of cholesterol crystals that contribute to inflammation, a key driver of atherosclerotic pathology. Finally, the authors shine a light on signaling mechanisms that recently have been further explored, such as those in digestive exophagy and enhanced pinocytic activity, which despite key differences, are similar, and may occur simultaneously.

Despite the use of lipid-lowering drugs, such as statins, ezetimibe, and fibrates, the residual cardiovascular risk remains high. It is imperative to find novel lipid-lowering agents and initiate a combination of lipid-lowering drugs with different but complementary mechanisms to lower residual lipid risk. PCSK9 is an enzyme that is mainly produced by the liver and subsequently released into circulation. PCSK9 cleaves the LDL-receptor (LDLR) protein expressed on hepatocytes, thereby precluding clearance of LDL cholesterol. Thus, PCSK9 inhibitors can promote LDL cholesterol by preventing LDLR degradation. Numerous studies have supported that PCSK9 levels are elevated in different settings of cardiovascular diseases. PCSK9 monoclonal antibodies and liver-targeted siRNAs have been approved to treat patients with hypercholesterolemia. Ma et al. recently reviewed the regulation of PCSK9 by transcription factors such as SREBP2, HNF-1α, and FOXO3. The authors also summarized the effects of PCSK9 in regulating lipid metabolism, inflammation, thrombosis, and cell death. Lastly, the relationship between PCSK9 and various forms of cardiovascular diseases, including atherosclerosis and myocardial infarction, was reviewed. This Review provides a timely overview of the role of PCSK9 in cardiovascular diseases and will provide a good reference for PCSK9-targeted therapies.

Diabetes (both type 1 and type 2) increases the risk of the development of atherosclerotic cardiovascular disease with different mechanisms. The existence of hyperglycemia induces, among others, exaggerated oxidative stress, inflammation, and ER stress, which act in concert to promote foam cell formation. Within the local atherogenic microenvironment, several different cell types, such as monocytes, macrophages, and vascular smooth muscle cells, can engulf modified LDL (oxLDL in particular) as well as triglyceride-rich lipoproteins to become foam cells. A recent Mini Review by Cervantes and Kanter discusses the role of monocyte and macrophage foam cells in the progression of diabetes-accelerated atherosclerosis by focusing on lipid uptake (via scavenger receptors CD36, SR-A, and LOX-1), cholesterol esterification, and lipid efflux (via efflux transporters ABCA1, ABCG1, and SR-B1) pathways. This Mini Review provides an important basis for foam cell-targeted therapies for treating diabetic atherosclerosis and its accompanying complications, such as diabetic kidney diseases.

This Research Topic, *Novel Strategies for the Prevention and Treatment of Foam Cell Formation and Atherosclerosis*, provides a brief indication of the current advancement and present-day questions related to the current understanding of foam cell formation and atherosclerosis. We, the editors, hope that this special issue will foster future research allowing the field to apply the best evidence-based interventions to combat cardiovascular diseases.
